# Diagnosing *P. simium* zoonotic malaria infection in the Rio de Janeiro Atlantic forest, Brazil

**DOI:** 10.1038/s41598-025-07900-y

**Published:** 2025-07-03

**Authors:** Maria de Fátima Ferreira-da-Cruz, Natália Ketrin Almeida-de-Oliveira, Rebecca de Abreu-Fernandes, Aline Rosa de Lavigne Mello, Graziela Zanini, Filipe Vieira Santos de Abreu, Anielle de Pina-Costa, Ricardo Lourenço-de-Oliveira, Patrícia Brasil, Cláudio Tadeu Daniel-Ribeiro

**Affiliations:** 1 Laboratório de Pesquisa em Malária, Instituto Oswaldo Cruz (IOC), Fundação Oswaldo Cruz (Fiocruz), Rio de Janeiro, RJ 21040-900 Brazil; 2 Centro de Pesquisa, Diagnóstico e Treinamento em Malária (CPD-Mal), Reference Laboratory for Malaria in the Extra-Amazonian Region for the Brazilian Ministry of Health, Secretaria de Vigilância em Saúde e Ambiente (SVSA) and Fiocruz, Rio de Janeiro, 21040-900 Brazil; 3 Instituto Nacional de Infectologia Evandro Chagas, Fiocruz, Rio de Janeiro, RJ 21041-361 Brazil; 4 Instituto Federal do Norte de Minas Gerais, Salinas, MG 39560-000 Brazil; 5 Universidade Federal Fluminense, Niterói, RJ 24220-900 Brazil; 6 Laboratório de Mosquitos Transmissores de Hematozoários, IOC, Fiocruz, Rio de Janeiro, RJ 21040-900 Brazil

**Keywords:** Malaria, Diagnosis, *P. vivax*, *P. simium*, Parasite genetics, DNA sequencing, Diagnosis

## Abstract

Malaria remains a pressing health challenge in Brazil, primarily due to *Plasmodium vivax*. Most cases occur in the Amazon; however, outbreaks in the Atlantic Forest involving *P. simium*, a non-human primate parasite closely related to *P. vivax*, have posed challenges for control efforts. This study aimed to differentiate *P. vivax* infections from those of *P. simium* acquired within the Atlantic Forest. Ninety-nine samples initially identified as *P. vivax*-positive, comprising 95 from humans and four from non-human primates, were analyzed. The results using a refined molecular tool revealed that 93% of human samples from the Atlantic Forest were *P. simium*, corroborating the substantial rate of zoonotic transmission in this region. The remaining samples from the Amazon and nearby countries were confirmed as *P. vivax*, including five Atlantic Forest cases linked to travel to endemic areas, suggesting imported or relapsed cases. The study emphasizes the importance of molecular tools in accurately distinguishing malaria parasite species, especially in identifying the origins of the parasites infecting humans. This understanding is crucial for grasping malaria transmission dynamics, ensuring accurate epidemiological surveillance, and preventing zoonotic transmission. It also supports targeted control strategies and effective public health interventions.

## Introduction

Malaria persists as a pressing public health concern in Brazil, particularly in the Amazon region, where *P. vivax* dominates as the leading cause of human infections^1–3^. Malaria is caused by parasites of the genus *Plasmodium and* transmitted by female-infected *Anopheles* mosquitoes. The most lethal parasite is *P. falciparum*, which predominates in Africa. *P. vivax* is prevalent in Asia and Latin America. *P. vivax* has an extensive geographic distribution worldwide, causing significant morbidity in Southeast Asia, the Americas, and the Middle East^1^.

In the Americas, 72% of malaria cases are attributed to *P. vivax*, with three countries—the Bolivarian Republic of Venezuela, Brazil, and Colombia—accounting for approximately 80% of the estimated cases in this region in 2023^1^. In Brazil, 138.327 malaria cases were reported in 2024, with 99.9% originating from states within the Legal Amazon region (Acre, Amapá, Amazonas, Maranhão, Mato Grosso, Pará, Rondônia, Roraima, and Tocantins), and around 82% of these cases were attributed to *P. vivax*^2^.

Despite ongoing control efforts over the years, malaria remains a significant health concern in Brazil, primarily due to the potential clinical severity of falciparum malaria. Furthermore, vulnerable populations living in poor housing and sanitation conditions experience considerable social and economic losses^3^.

Brazil has a diverse geographical profile that leads to variations in malaria transmission ecology, with regions divided into three groups, each featuring unique transmission settings^4^. First, the endemic Brazilian Amazon (BA) rainforest in northeastern Brazil accounts for more than 99% of all malaria cases, primarily driven by *Anopheles (Nyssorhynchus) darlingi*. Second is Brazil’s coastal lowland border, particularly in the northeast, where transmission has been lower, typically due to an epidemic of *(Nys.) aquasalis*. Third, the Atlantic Forest (AF) mainly extends along the southern and southeastern Atlantic coast, where transmission is significantly lower and primarily facilitated by *An. (Kerteszia) cruzii*, known as bromeliad malaria^4,5^. Female mosquitoes of the subgenus *Kerteszia* lay their eggs in water accumulated in phytotelmata, predominantly in bromeliads, which are abundant in the AF biome^6,7^.

Malaria is a notifiable disease in the Amazon region and must be reported to Brazilian health authorities within 7 days. In contrast, in the extra-Amazonian region, malaria is an immediate compulsory notification disease; that is, every suspected case must be notified to health authorities within 24 h by the fastest means available^8^.

In 2015, an unexpected outbreak of malaria occurred in the AF, specifically in the southeastern state of Rio de Janeiro (RJ), with the identification of 33 autochthonous cases, all acquired in mountain areas in the AF. This marked more than a fourfold increase compared to the average number of annual autochthonous cases in the state, which accounted for 40% of all autochthonous cases outside the endemic area in the country. The infections were caused by the non-human parasite *P. simium*^9^. *P. simium* is a malaria parasite affecting the *Platyrrhini* monkey species in the AF biome of Southeast and South Brazil^6^. *P. simium* shares genetic, morphological, and immunological similarities with cosmopolitan *P. vivax*^9^. This acute febrile infectious disease poses a significant public health issue. However, control measures have primarily targeted urban areas, neglecting the dense AF where silent *Plasmodium* reservoirs exist^6^. Given that *P. simium* is quite like *P. vivax* and that these infections are confined to the AF biome, we evaluated the malaria cases that occurred over the last years in the region to identify the species causing the infections through 2 SNPs that differentiate *P. simium* from *P. vivax* in the parasite’s mitochondrial DNA^9^. Therefore, to understand transmission dynamics in the region and help in decision-making to implement more effective control strategies, we evaluated a nested PCR^10^ combined with DNA sequencing to differentiate *P. vivax* endemic from zoonotic *P. simium* malaria infections.

## Results

Among the 99 samples screened to amplify the cox1 mitochondrial gene, 95 were from humans (69 AF, 25 BA, and 5 Brazilian bordering countries - BBC), and four were from howler monkeys, all of which were successfully sequenced. The fragment of the cox1 mitochondrial gene was sequenced to differentiate *P. simium* from *P. vivax* by identifying two single-nucleotide polymorphisms (SNPs) located in T3535**C** and A3869**G** for *P. simium*^9^. All 25 human *P. vivax* samples from the Amazonian states [Acre (2), Amapá (3), Amazonas (17), Pará (1), Rondônia (1), and Roraima (1)] as well as the five non-Brazilian samples (BA), showed the cox1 mitochondrial gene sequences identical to the reference *P. vivax* SAL-1 (Table 1). No sequences with mixed genotypes of SNPs corresponding to the *P. vivax and P*. simium parasites could be detected.

**Table 1:** Frequency of *P. simium* specific single-nucleotide polymorphisms (SNPs) in human and non-human primate isolates, based on sampling locality.

Locality (*N* of samples)	Sample type	3535T *P. vivax* (NP/%)	3535C *P. simium* (NP/%)	3869C *P. vivax* (NP/%)	3869G *P. simium* (NP/%)
Atlantic forest (*n* = 69)	Human	5/7	64/93	5/7	64/93
Amazon rainforest (*n* = 25)	Human	25/100	0	25/100	0
Brazilian bordering countries (*n* = 5)	Human	5/100	0	5/100	0
Ilha Grande, RJ (*n* = 2)	NHP	0/0	2/100	0/0	2/100
Morro São Pedro, RS (*n* = 2)	NHP	0/100	2/100	0/100	2/100

The characters in red and underlined indicate the nucleotide substitutions; NP: number of positive; NHP: non-human primate; RJ: Rio de janeiro; RS: Rio Grande do sul.

**Fig. 1:**
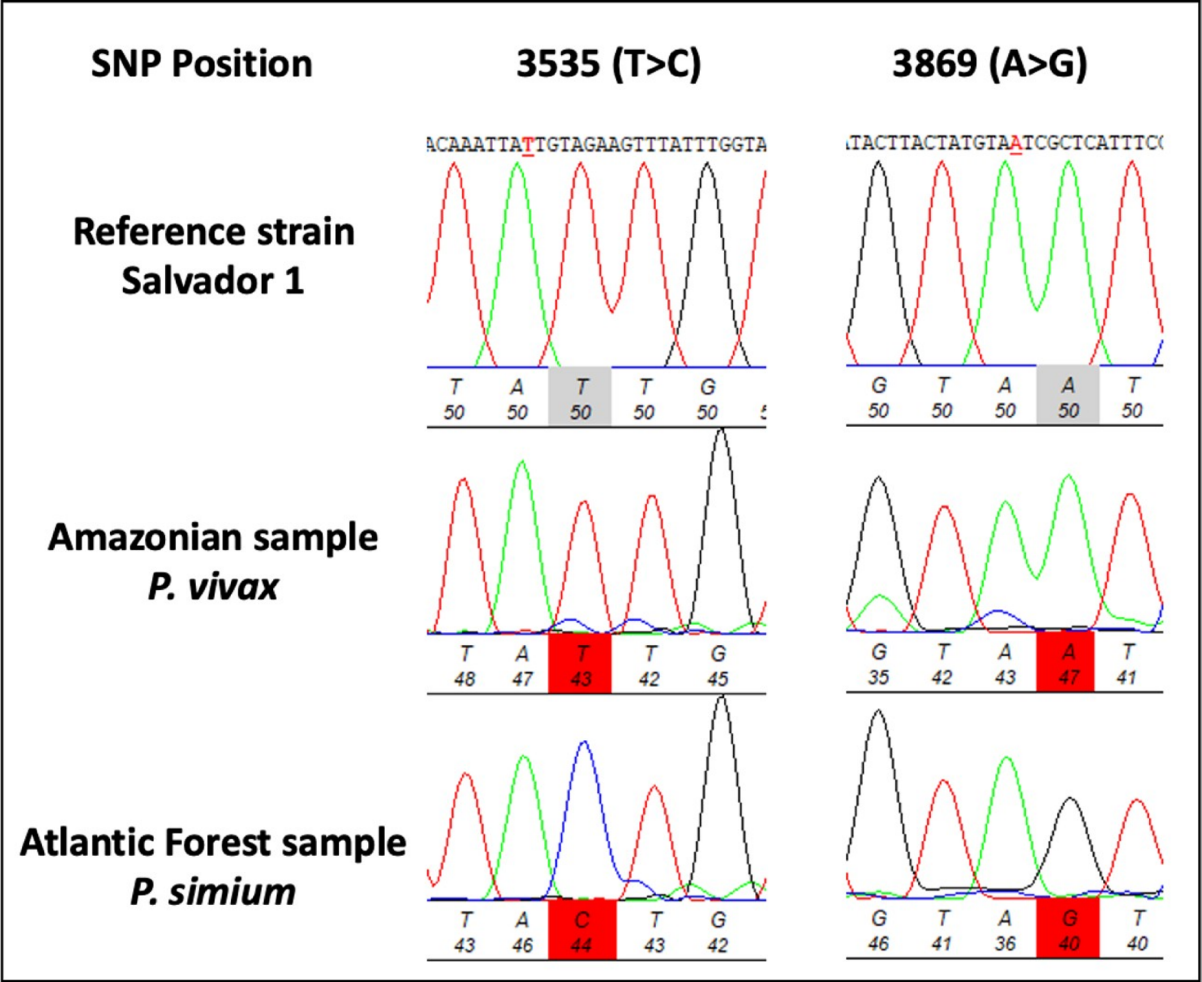
Brazilian map highlighting the states and municipalities affected by parasitic infection. This map was generated using ArcGIS Online. https://www.arcgis.com/apps/mapviewer/index.html.

**Fig. 2:**
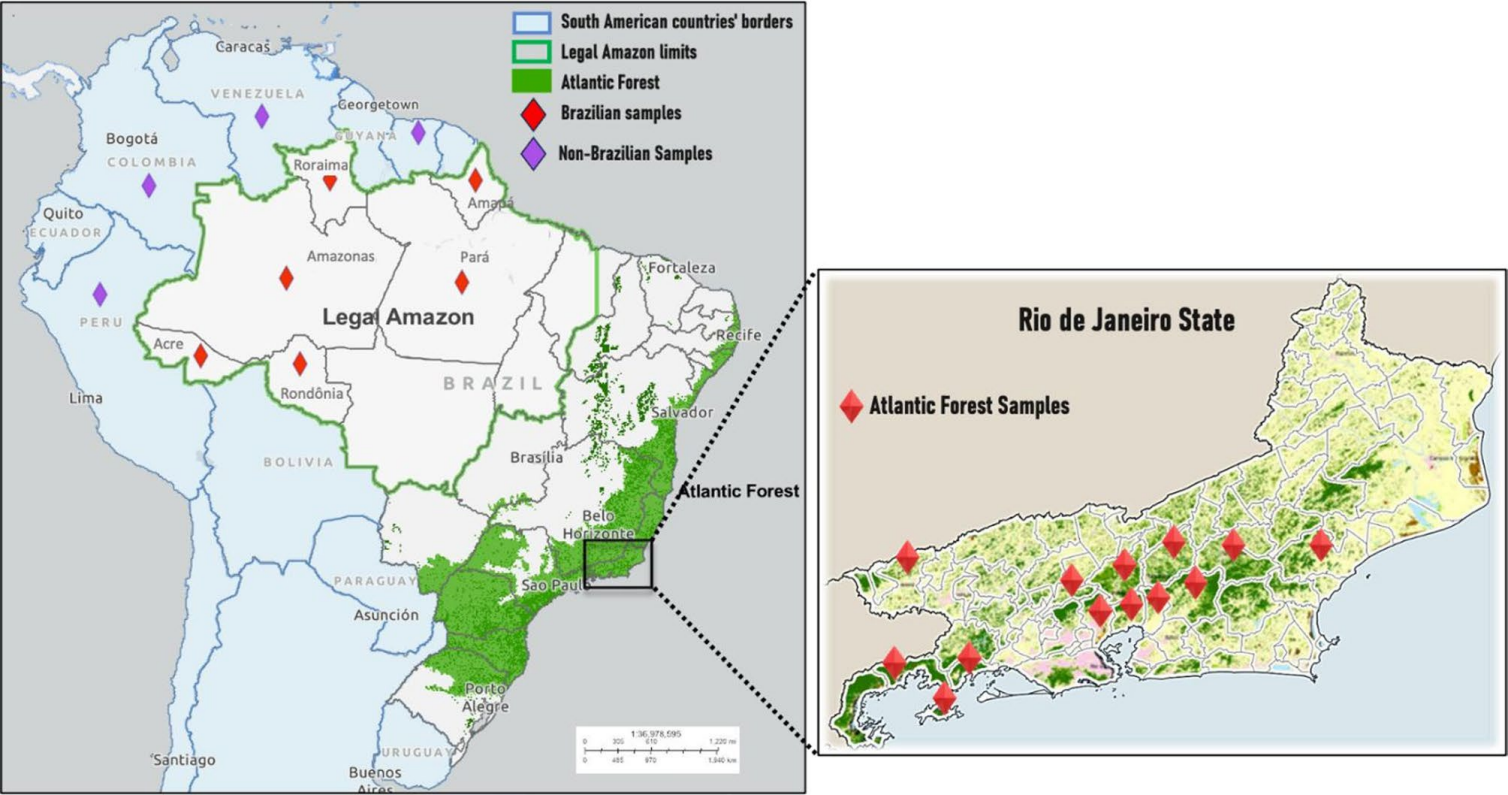
Electropherogram highlights *P. simium*-Specific SNPs in Amazon and Atlantic Forest, Brazil samples. The characters in red and underlined represent the nucleotide substitutions in the isolates.

Among the 69 human blood isolates collected from patients in the AF, 64 (93%) presented the two *P. simium*-specific SNPs (Fig. 1), and five (7%) had their sequences identical to those of *P. vivax* in the Amazonian region. All 64 samples exhibiting the two *P. simium*-specific SNPs were from people who live or have visited the mountain valleys in the AF, where *An. cruzii* is the predominant anopheline species (Fig. 2).

Five AF isolates (two in 2011, one in 2016, and two in 2017) showed similar amino changes compared to BA isolates (*P. vivax*). When an epidemiological investigation was conducted to characterize their possible infection location, it was discovered that all of them had traveled to or were primarily residents of malaria-endemic areas or lived in the lowlands of RJ where *Nyssorhynchus* mosquitoes predominate and *An. cruzii* is absent or rare. Two patients who have traveled to the Brazilian Amazon malaria-endemic area before symptoms could be classified as *P. vivax* imported cases. The other three patients lived in malaria-endemic places before moving to Rio de Janeiro (two in the Brazilian Amazon and the other in Equatorial Guinea), suggesting that these were relapse *P. vivax* malaria cases by hypnozoites.

The four samples from howler monkeys originate from two Brazilian states in the AF, Rio de Janeiro, and Rio Grande do Sul, presented the two *P. simium*-specific SNPs (Table 1).

## Discussion

*P. knowlesi* and *P. cynomolgi*, classical non-human primate (NHP) malaria parasites in Southeast Asia, exemplify the recent emergence of zoonotic transfer to humans^11,12^. The competence of appropriate mosquito vectors that bite both humans and NHP hosts, along with globalization, climate change, and increased human encroachment into monkey habitats, are key factors driving the emergence of simian malaria parasites in humans.

Thus, failure to distinguish *P. simium* from *P. vivax* risks underestimating zoonotic transmission and obscuring transmission dynamics in regions like the AF, where human-primate interactions are frequent^3,9^. Sensitive and specific molecular tools for individual diagnosis and epidemiological studies can help avoid this risk. The epidemiology of non-Ps/Pv species in areas under the influence of the AF is still understudied.

Importantly, platyrrhine monkeys and humans share mitochondrial lineages of the *P. vivax*-like species *P. simium* in the AF ecosystem of South and Southeast Brazil but not in the Amazon^13^. Although *P. vivax* is responsible for most malaria cases in Brazil, mainly in the Amazon, the equivalent species, *P. simium*, has never been found infecting monkeys outside the south and southeast areas of AF^14,15^. Here, we confirmed that the lineages circulating in humans and monkeys in southern and southeastern Brazil differ by at least two single-nucleotide substitutions at the cox1 *locus* A3325T and A3869G^9,16,17^.

Recently, our collaborators developed an essay to identify the infection sources of *P. simium* using nested PCR associated with RFLP^10^. RFLP refers to a type of polymorphism caused by variations in DNA sequences recognized by restriction enzymes. RFLP is simpler and cheaper than sequencing PCR products, but is also slower and more tedious than newer DNA analysis methods, such as DNA sequencing. Common issues encountered during RFLP analysis, such as lack of digestion, poor-quality digestion, unexpected digestion, and over-digestion, can lead to misleading results.

In the present study, we report the molecular detection and characterization of *P. simium* found in blood samples from humans and monkeys initially identified as positive for *P. vivax* through microscopy and PCR. Based on BLAST analyses of the *cox1* mitochondrial gene, the samples were confirmed to be *P. simium*. Among the 69 patients evaluated who were living in or visiting areas influenced by the AF, 64 (96%) were infected with *P. simium*. The infections in these individuals may have been transmitted by *An. cruzii*, the predominant anopheline in the mountain areas where they reside or visit. It has been shown to bite humans at ground level and monkeys in the forest canopy.

Our analysis demonstrated a significant diagnostic challenge in distinguishing between *P. vivax* and *P. simium*parasites in Brazil, as 93% of human cases initially identified as *P. vivax* in the AF contained *P. simium*-specific SNPs. This underscores the complexity of malaria transmission in areas where humans and non-human primate hosts coexist. Adaptation to various local mosquito vectors may have driven the divergence between *P. vivax* lineages introduced to Brazil, which now exist in distinct, non-contiguous endemic *foci* in the Amazon (*P. vivax*) and along the Atlantic Coast (*P. simium*).

## Conclusion

If not addressed promptly, emerging zoonotic malaria species such as *P. simium* could jeopardize efforts to eliminate malaria. The assay described here corresponds to one more effective tool for accurately diagnosing malaria cases in the AF.

## Methods

The work was carried out at the *Laboratório de Pesquisa em Malária*, headquarters of the *Centro de Pesquisa*,* Diagnóstico e Treinamento em Malária (CPD-Mal*), the National Reference Centre for Diagnostics and Training in the extra-Amazon Region located at the *Fundação Oswaldo Cruz (Fiocruz)*, Rio de Janeiro, southeast Brazil.

### Study population

A total of 99 *P. vivax*-positive PCR human isolates were analyzed at the *Laboratório de Pesquisa em Malária*. The samples were separated according to the local of infection: Rio de Janeiro AF (69), BA Rainforest (25), and BBC as Colombia (1), Peru (1), Suriname (1), and Venezuela (2). In addition, samples of four howler monkeys - *Alouatta guariba clamitans -* naturally infected with *P. vivax*-like monkey *P. simium* parasites in AF were also tested. The *P. simium / P. vivax* diagnosis was made by light microscopy (thick and thin blood smears Giemsa-stained slides) followed by *P. vivax* PCR^8,19^. Only patients with *P. vivax* mono-infections were included in the study.

### Ethical aspects

Human venous blood collection was performed after obtaining written informed consent from all participants, in accordance with protocols approved by the Ethical Research Committees of Fiocruz (CAAE 88554718.0.3002.5248). All participants were informed about the study and signed a written consent form prior to enrollment.

Monkey collection procedures were approved by the Institutional Ethics Committee for Animal Experimentation of Instituto Oswaldo Cruz (CEUA/IOC, protocol L-037/2016), the Brazilian Ministry of the Environment (SISBIO 54707–4), and the Environment Agency of Rio de Janeiro (INEA 012/2016 and 019/2018). This study is reported in accordance with the ARRIVE guidelines (https://arriveguidelines.org).

All methods involving human participants and animals were conducted following the relevant institutional guidelines and regulations.

### DNA extraction, amplification, and sequencing

Human DNA was extracted from 1 mL of whole blood samples using the QIAamp™ DNA Blood Midi Kit (Qiagen, Hilden, Germany), according to the manufacturer’s instructions and those for non-human primates, following a protocol developed by our group^20^. *P. vivax*-positive samples were initially identified through molecular diagnosis using Real-time^18^ and conventional PCRs^19^. Since these PCRs do not distinguish cosmopolitan *P. vivax* from *P. simium*, we amplified the cox1 mitochondrial gene. The amplified fragment contains two unique single-nucleotide polymorphisms (SNPs) at positions 3535 (T > C) and 3869 (A > G), which are found only in *P. simium*^9^.

The protocol was conducted as previously described, with some modifications. Briefly, a single PCR reaction was performed using the primer pair psimOUTF 5′CAGGTGGTGTTTTAATGTTATTATCAG3′ (forward) and psimINR 5′ATGTAAACAATCCAATAATTGCACC3′ (reverse) in a 20 µL reaction volume containing 1 µM of each primer, 5x HOT FIREPol™ Blend Master Mix (Solis Biodyne, Hannover, Germany) that has high fidelity and proofreading activity, 2 µL (100–200 ng) of DNA, and nuclease-free water. The amplification thermal cycling conditions included an initial hold at 94 °C for 12 min, followed by 30 cycles of 94 °C for 30 s, 54 °C for 30 s, and 72 °C for 30 s, concluding with a final hold at 72 °C for 7 min. Human positive and negative controls, along with a non-template control, were included in each amplification test. PCR products were run on a 2% agarose gel, stained with ethidium bromide, and the specific 567-base pair band was visualized using a UV light transilluminator (DigiDoc-It, UVP, California, USA). DNA templates were purified using the Wizard™ SV Gel and PCR Clean-Up System (Promega, Wisconsin, USA), following the manufacturer’s protocol, and then sequenced with Big Dye™ Terminator Cycle Sequencing Ready Reaction version 3.1 (Applied Biosystems, California, USA), using 3.2 µM of forward and reverse PCR primers on an ABI Prism DNA Analyzer™ 3730 sequencer (Applied Biosystems, California, USA) at the Fiocruz Genomic Platform PDTIS/Fiocruz RPT01A.

### Data analysis

Nucleotide sequences were aligned using the Clustal W multiple sequence aligner in the BioEdit™ version 7.7.1 software^21^ (North Carolina State University, Raleigh, NC, USA, and the electropherogram was set to a 10-cutoff score using NovoSNP software^22^. Sequences were deposited in the GenBank™ database (NIH genetic sequence database; www.ncbi/nlm/nih.gov/GenBank) under accession numbers OR124630-OR124730. The reference sequences were the Salvador-1 strain of *P. vivax* (accession number plasmoDB PVAD80_MIT https://plasmodb.org/) and *P. simium* (accession number plasmoDB NC_007233.1https://plasmodb.org/).

## Data Availability

Data supporting the conclusions of this article are included within the article. The datasets used and/or analyzed during the present study are available from the corresponding author upon reasonable request.
